# Case Report on Pleural Empyema Thoracis and Urinary Tract Infection Caused by *Chromobacterium violaceum* from Lagos, Nigeria

**DOI:** 10.1155/2019/5321484

**Published:** 2019-02-07

**Authors:** Adesola Olalekan, Faith Itua, Bamidele Mutiu, Tenny Egwuatu, Olubukola Akinloye, Bamidele Iwalokun

**Affiliations:** ^1^Department of Medical Laboratory Science, College of Medicine, University of Lagos, Lagos, Nigeria; ^2^Department of Medical Microbiology & Parasitology, Lagos State University Teaching Hospital, Lagos, Nigeria; ^3^Department of Microbiology, Faculty of Science, University of Lagos, Lagos, Nigeria; ^4^Department of Medical Microbiology & Parasitology, Olabisi Onabanjo University, Ago Iwoye, Nigeria; ^5^Molecular Biology & Biotechnology Department, Nigerian Institute of Medical Research, Lagos, Nigeria

## Abstract

*Chromobacterium violaceum* has been implicated as an important cause of invasive diseases such as septicaemia in neonates and immune-compromised adults with high risk of misdiagnosis, mistreatment, and poor outcomes. Here, we report three new cases of *C. violaceum* infections in three different hospitalised patients with empyema thoracis (one case) and urinary tract infections (two cases) in a tertiary Hospital in Lagos, Nigeria, and the diagnosis was confirmed with the MALDI-TOF MS instrument. The patients were admitted and treated with parenteral antibiotics (ciprofloxacin, cefotaxime, and ceftriaxone) and discharged after clinical cure. Clinical and Laboratory findings from this study revealed *C. violaceum* as an emerging and an “underdiagnosed” pathogen causing human infections in Nigeria with ciprofloxacin identified as an effective empirical treatment. Follow-up of cases treated with microbiologically efficacious antibiotics indicates a good treatment outcome.

## 1. Introduction

*Chromobacterium violaceum* is a Gram-negative, facultative anaerobic, non-spore-forming, motile, oxidase-positive coccobacillus. Its main reservoirs are water and soil of tropical and subtropical regions. The organism produces violacein, a natural compound with industrial applications in pharmacology and cosmetics [1]. *C. violaceum* is an opportunistic pathogen, and the infection can start as a mild skin infection that eventually progress into necrotizing or metastatic lesions. Multiple abscesses can occur in solid organs (e.g., liver, lung, spleen, and brain), leading to multiorgan failure [2–7]. *C. violaceum* diseases can occur suddenly and could severely advance into a life-threatening situation [8]. It has been established that treatment is achievable with classes of antibiotics such as quinolones and carbapenems [9]. *C. violaceum* was first reported as a pathogen in humans from Malaysia in 1927 [10]. Since then, about 150 cases have been reported globally [6]. In Africa, cases of *C. violaceum* have been reported in Congo (bacteraemia) [11], Senegal (diarrhea) [12], and South Africa (sepsis) [13].

In Nigeria, two studies by Onile et al. [14] and Anah et al. [15] have also documented the occurrence of *C. violaceum* infections among hospitalised patients in north central and South Eastern Nigeria in wound infection and neonatal septiceamia.

One of the medical challenges posed by *C. violaceum* is its misidentification as *Burkholderia pseudomallei* when conventional microbiological methods are used [16]. This could increase the risk of inappropriate antibiotic treatment and medical management resulting in poor prognosis and unfavourable treatment outcome.

Here, we report three cases of a *C. violaceum* infection from patients admitted to Lagos State University Teaching Hospital (LASUTH) Ikeja from March 2014 to June 2016. Species of *C. violaceum* was isolated from pleural effusion and urine samples, identified conventionally using the purple pigment morphology, oxidase test, and sugar fermentation, and subsequently confirmed with matrix-assisted laser desorption/ionization/time-of-flight (MALDI-TOF) in Münster (microflex LT; Bruker Daltonik, Bremen, Germany), using the MALDI Biotyper library (Version 3.3.2.0). Antibiotic susceptibility was tested using the Kirby Bauer disc diffusion method, with seven different antibiotics disks (Oxoid, Basingstoke, UK): ciprofloxacin (5 *µ*g), amoxicillin/clavulanate (20/10 *µ*g), cefotaxime (30 *µ*g), gentamicin (15 *µ*g), ceftriaxone (30 *µ*g), trimethoprim/sulfamethoxazole (1.25/23.5 *µ*g), and imipenem (30 *µ*g) according to the recommendation of the Clinical Laboratory Standards Institute (CLSI) M100-S24. The inhibition zone diameter breakpoints for enterobacteriaceae were used to assess the antibiotic susceptibility.

## 2. Case Presentation

### 2.1. Case 1

The patient (31 years old, male) presented with body weakness, anorexia, and headache at the surgical emergency service department. The patient was febrile (axillary body temperature, 37.9°C) and had a heart rate of 98 beats/min. He was tachypnoeic with a respiratory rate of 40/min and normal blood pressure of 110/70 mmHg. The patient had initially presented two months earlier with a history of unproductive cough that was of insidious onset, and cough was intermittent in nature. He had fever, malaise, and difficulty with breathing. There was no orthopnoea, paroxysmal nocturnal dyspnoea, or a history of contact with persons with a chronic cough. Later, the patient observed that there was difficulty in breathing soon after the onset of a cough that worsened with moderate activities such as climbing a staircase and also a significant weight loss. On further clinical examination, the chest was asymmetrical, with reduced chest expansion and tactile fremitus on the left lung field. There was stony dullness to percussion and reduced vocal resonance over the same area. There was also reduced air entry in the left lung field. Other systems were essentially within the normal limit. Chest radiograph showed a massive left pleural effusion and deviation of the trachea to the right. Ultrasonography scan showed a massive left-sided pleural effusion with lung abscess. The differential diagnoses were lobar pneumonia complicated by pleural effusion. The patient had a closed thoracotomy tube drainage with an initial drainage of 600 ml of pus. A Ziehl–Neelsen staining of the pleural effusion showed no acid-fast bacilli, and the final diagnosis was empyema thoracis.

The hematogram and the clinical chemistry laboratory results are shown in Table 1.

The microbiological culture of the pus from the pleural empyema showed a culture of *C. violaceum* on Columbia blood agar and MacConkey agar. *C. violaceum* was oxidase positive, indole negative, utilized citrate, catalase positive, and fermented malonate. *C. violaceum* was susceptible to ciprofloxacin, gentamicin, amoxicillin/clavulanic acid, and imipenem. Resistance was detected against ceftazidime, cefuroxime, and cotrimoxazole.

He was treated empirically with intravenous amoxycillin-clavulanic acid and ceftriaxone for seventy-two hours. Due to poor patient response, once we had the sensitivity result from the microbiology examination. He was switched to intravenous ciprofloxacin 200 mg 12 hourly for one week and was discharged after clinical cure. He was treated for one week with intravenous ciprofloxacin as an inpatient.

### 2.2. Case 2

An infant (two months old, male) was admitted to the paediatric unit of the hospital on account of fever, convulsion, and vomiting. There was no other significant past medical history prior to presentation. The family history indicates that the family lives in an one-room apartment with a borehole as the sole source of water. The two-month-old baby was exclusively breastfed as per the national policy on breastfeeding of babies in the first 6 months of life. The urine stream was normal, and the flow was adequate. There was no posterior urethral valve: this would have been discovered in the first week of life before discharge from the hospital after birth since all the male infants are always examined before discharge. The convulsion in this patient is unlikely to be a febrile convulsion; unfortunately, EEG was not done which would have been helpful in differentiating between seizures and abnormal movements. Clinical examination showed no abnormality (axillary temperature, 36.7°C, heart rate: 152 beats/min, respiratory rate: 22/min, weight: 5.8 kg; an electrocardiogram carried out was also normal). But occipitofrontal circumference is 44.4 cm, and this is macrocephaly which should have been investigated further. Other physical examinations were within the normal limits. Clinical Laboratory investigation findings are as given in Table 1.

CSF culture did not show bacterial growth. A clean-catch, amber-coloured urine was collected, nonturbid with a pH of 6.5, and had a specific gravity of 1.005; all other urine parameters such as leukocyte and protein were negative.

Urine culture yielded the growth of *C. violaceum* (5.5 × 10^8^ CFU/ml). The antibiotic susceptibility testing showed sensitivity to ofloxacin, ciprofloxacin, nitrofurantoin, amoxicillin/clavulanic acid, cefuroxime, ceftazidime, imipenem, and gentamicin. The final diagnosis was urinary tract infection. The patient was treated with intravenous cefotaxime (275 mg, bd for 7 days) and was later discharged after clinical cure, as part of the national guidelines; the child initially received intramuscular artemether (Paluther®) until malaria was ruled out.

### 2.3. Case 3

A child (5 years old, male) was admitted at the children ward of the hospital on account of fever and generalised oedema, and no sign of malnutrition was noticed. The patient was not in daily contact with farmland or stagnant water, and there was no history of any skin lesion or a sore throat prior to his admission to the hospital. The oedema was initially periorbital before becoming generalised. The parents did not observe any deviation in the frequency of passage of urine but the urine was frothy in nature. Clinical examination revealed a febrile child (38°C), not pale, with a body weight of 18 kg (45th percentile), and a height of 112 cm (67th percentile) [17]. The pulse rate was 96 beats/min, the blood pressure was 84/50 mmHg, and laboratory investigations results are given in Table 1.

The clean-catch urine was amber coloured with a pH of 6.0 and specific gravity of 1.020. The urine was positive for protein (++), leukocytes (++), and ketones (+) with only traces of blood (ACON Laboratories, San Diego, USA). The 24-hour urinary protein was 1.99 g/24 hours (normal range: <100 mg/24 hours) with a urine volume of 410 ml. Urine microscopy indicated pus cell of 3-4 cells per high power field. Urine culture yielded growth of *C. violaceum* (5.8 × 10^8^ CFU/ml), which was susceptible to ofloxacin, ciprofloxacin, gentamicin, nitrofurantoin, and imipenem and resistant to amoxicillin/clavulanic acid, ceftazidime, cefuroxime, and cotrimoxazole.

The patient received intravenous ceftriaxone (500 mg, 12 hourly, for 7 days) until the temperature became normal; furosemide (iv) dose of 40 mg 8 hourly for the first 24 hours was later reduced to 20 mg 8 hourly for four days and then switched to oral furosemide at a dose of 20 mg tds for 3 days with spironolactone (po, 12.5 mg bd for 7 days). Spironolactone is used to give the additive potassium ion-sparing effect. The patient's symptoms regressed with treatment. The patient received two pints of blood due to anemia. The final diagnosis was urinary tract infection, and the patient was discharged home after clinical improvement for follow-up two weeks later. The quick recovery of this patient and the normal urinalysis at the hospital follow-up visits were not in conformity with nephrotic syndromes (differential diagnosis). The fact that the symptoms regressed without dialysis and no relapse on follow-up are enough to rule out chronic renal failure.

## 3. Discussion

*Chromobacterium violaceum* is an emerging bacterial infection in Nigeria. We described three cases of *C. violaceum* infections associated with empyema thoracis (one case) and urinary tract infection (two cases). Bacteriology examination of the aetiology of infections in Nigeria most times lacks molecular techniques to confirm *C. violaceum*. Misdiagnosis may not be ruled out since most laboratories do not focus on this organism; they only use available laboratory techniques to isolate the common organism, and when found, they may not be able to either recognise or identify the organism accurately. The organism can also be misdiagnosed with *Burkholderia pseudomallei* [16]. We used the standard conventional method in our laboratory to identify the organism. The organism was further confirmed by MALDI-TOF, a more advanced proteomic tool. *C. violaceum* septicaemia can be fatal even in adults [11]. Proper identification and reporting of this organism are therefore essential for the clinician to develop an appropriate treatment strategy for the patients' management. This information is also vital for the hospital infection control team to identify possible environmental (e.g., water and formites) and human sources of *C. violaceum*. Inadequate documentation is a barrier to be able to associate other clinical factors to the infection in this study. It is worth noting that all these patients did not have access to farmland, and the source of their drinking water was boreholes. *C violaceum* is commonly known to be resistant to a cephalosporin [6], but the UTI cases in our study was treated with cephalosporin; minimum inhibitory concentration (MIC) data could have produce a clearer picture, but this was not performed.

The predominance of male sex and young age among our cases is consistent with a systematic clinical review that included Americans and South Asians [7]. Two of our cases were underage, who were known to have a less matured immune system compared with that of the adults and chronic illness in case of the patients with empyema thoracis; he had been unwell for about months before diagnosis; this confirms *C. violaceum* as an opportunistic infection. The two previous cases of *C. violaceum* in Nigeria [14, 15] were reported for wound infection and neonatal septicaemia from two different geographical regions in the country that are more than 1000 km apart. The present study has further revealed the clinical heterogeneity of *C. violaceum* as an opportunistic pathogen with the ability to cause empyema thoracis and UTI as well as that demographically affects all age groups. Therefore, further characterisation of *C. violaceum* strains in Nigeria is a necessity so as to have detailed epidemiological data on risk factors, ecology, virulence, antimicrobial resistance genes, and clonal structure for effective prevention and halting of its transmission in this region.

## 4. Conclusion

*Chromobacterium violaceum* is an emerging and an “underdiagnosed” pathogen causing serious human infections in Nigeria. Conventional identification and supportive molecular identification of *C. violaceum* can be helpful in the differential diagnosis of drug-resistant Gram-negative bacteria causing UTI and empyema thoracis. Empirical parenteral ciprofloxacin and ceftriaxone were effective in the treatment of *C. violaceum* infection.

## Figures and Tables

**Table 1:** Clinical and laboratory findings.

Investigations	Case 1	Case 2	Case 3	Reference ranges
Packed cell volume	0.33 L/L	0.33 L/L	0.20 L/L	0.37–0.47 L/L (case 1) 0.30–0.43 L/L (cases 2 and 3)
Heamoglobin	NA	NA	60 g/L	110–165 g/L
White blood cell (WBC)	11.2 × 10^9^/L	15.6 × 10^9^/L	36.0 × 10^9^/L	3.5–11 × 10^9^/L
Differential WBC				
Neutrophil	80%	19%	87%	40–75%
Lymphocytes	16%	75%	10%	20–45%
Monocyte	4%	6%	3%	2–10%
Platelet count	532 × 10^9^/L	220 × 10^9^/L	ND	150–400 × 10^9^/L
Erythrocyte sedimentation rate (Westergreen method)	85 mm in 1 hour	ND	ND	0–4 mm in 1 hour
Haemoglobin genotype	AA	ND	ND	ND
Blood group A+	A+			
HIV screening	Negative			
Urea	3.9 mmol/L	1.3 mmol/L	12.2 mmol/L	1.7–8.3 mmol/L
Sodium	129 mmol/L	131 mmol/L	130 mmol/L	133–150 mmol/L (case 1) 138–145 mmol/L (cases 2 and 3)
Bicarbonate	22 mmol/L	18 mmol/L	16 mmol/L	18–22 mmol/L
Creatinine	61.9 mmol/L	44.2 mmol/L	141 mmol/L	44.2–97 mmol/L
Potassium	3.8 mmol/L	5.1 mmol/L	2.8 mmol/L	3.5–5 mmol/L 3.4–4.7 mmol/L (cases 2 and 3)
Chloride	96 mmol/L	104 mmol/L	96 mmol/L	96–110 mmol/L
Cerebrospinal fluid	NA		NA	
Analysis		2.2 mmol/L		2.5–4.4 mmol/L
Glucose		6 g/L		15–45 g/L
Microprotein		30 cells/mm^3^		0–30 cells/mm^3^
CSF cell count		Mainly lymphocytes		
Total blood protein	NA	NA	37 g/L	65–81 g/L
Albumin	NA	NA	9 g/L	30–50 g/L
Globulin	NA	NA	28 g/L	20–35 g/L
Ziehl–Neelsen staining	No acid-fast bacilli seen	NA	NA	
Body temperature	37.9°C	36.7°C	38°C	
Heart rate	98 beats/min	152 beats/min	96 beats/min	
Respiratory rate	40/min	22/min	ND	
Blood pressure	110/70 mmHg	ND	84/50 mmHg	
Weight	NA	5.8 kg	18 kg	
Occipitofrontal circumference	NA	44.4 cm	NA	
Electrocardiogram	ND	Normal	NA	
Urine analysis	NA	Colour: amber Appearance: nonturbid pH 6.5 Specific gravity: 1.005 Leukocyte-negative Protein-negative	Colour: amber pH 6.0 Specific gravity: 1.020 Protein: ++ Leukocytes: ++ Ketone: + Blood: trace 24 hours urinary Protein = 1.99 g/24 hrs Urine volume = 410 ml Urine microscopy = pus cell 3-4HPF	Urinary protein ≤100 mg/24 hrs

NA: not applicable; ND: not done.
